# Transmission Pathways of Zoonotic Influenza Viruses and Influencing Factors: A Systematic Review of Recent Findings

**DOI:** 10.3390/v17060857

**Published:** 2025-06-17

**Authors:** Rebecca Badra, Wenqing Zhang, John S. L. Tam, Richard Webby, Sylvie van der Werf, Sergejs Nikisins, Ann Cullinane, Saad Gharaibeh, Richard Njouom, Malik Peiris, Ghazi Kayali, Jean-Michel Heraud

**Affiliations:** 1 Human Link DMCC, Dubai 0000, United Arab Emirates; rebecca@human-link.org; 2 Global Influenza Programme, World Health Organization, 1211 Geneva, Switzerland; zhangw@who.int (W.Z.); nikisinss@who.int (S.N.); 3 Department of Applied Biology and Chemical Technology, The Hong Kong Polytechnic University, Hong Kong SAR 999077, China; john.sl.tam@connect.polyu.hk; 4 Department of Infectious Diseases, St. Jude Children’s Research Hospital, Memphis, TN 38105, USA; richard.webby@stjude.org; 5 Molecular Genetics of RNA Viruses Unit, Institut Pasteur, 75015 Paris, France; sylvie.van-der-werf@pasteur.fr; 6 Virology Unit, The Irish Equine Centre, Johnstown, Naas, Co., W91 RH93 Kildare, Ireland; acullinane@irishequinecentre.ie; 7 Department of Veterinary Pathology and Public Health, Jordan University of Science and Technology, Irbid 22110, Jordan; saadgh@just.edu.jo; 8 Virology Unit, Centre Pasteur du Cameroun, Yaoundé P.O. Box 1274, Cameroon; njouom@pasteur-yaounde.org; 9 School of Public Health, The University of Hong Kong, Hong Kong SAR 999077, China; malik@hku.hk

**Keywords:** zoonotic influenza virus, cross-species transmission, transmission pathways, animal–human interface, transmission factors

## Abstract

Recent outbreaks of zoonotic influenza viruses underscored the need for a deeper understanding of transmission pathways and factors influencing spillover events. Understanding the combined effects of environmental conditions, host interactions, and viral adaptations is essential for effective preparedness and response. The WHO public health research agenda for influenza, revised in 2017, recommended research to further define the host-to-host transmission pathways of influenza type A viruses. Since 2017, important research has been conducted, and the global health landscape has changed. Therefore, there is a need to review the transmission pathway studies conducted during the last eight years. We conducted a systematic analysis following the PRISMA guidelines on 7490 PubMed records from 2017 to 2024, of which 219 records were retained. This review evaluates research on zoonotic influenza virus transmission among wild and domestic animals and cross-species transmission to humans. By examining pathways, host, environmental, and viral factors, this review identified key findings and research gaps. Research remains limited in critical areas including transmission pathways among diverse animals, role of environmental factors, and zoonotic potential across regions. Addressing these gaps is essential for improving public health strategies. This review highlights the necessity of integrating a One Health approach in addressing zoonotic influenza risks.

## 1. Introduction

Zoonotic influenza viruses, originating from avian or swine reservoirs, have significant implications for public health due to their capacity to adapt to human hosts and potentially cause epidemics and pandemics [1]. Influenza A viruses, which include the highly pathogenic avian influenza (HPAI) subtypes H5N1 and H7N9 and low pathogenic avian influenza (LPAI) subtype H9N2, can be transmitted across species barriers under conducive environmental and biological conditions [2]. Such cross-species transmission not only endangers human health but also impacts animal populations, agriculture, and economies worldwide [3]. Recent outbreaks of zoonotic influenza, such as those caused by H5N1, have underscored the need for a deeper understanding of transmission pathways and factors influencing spillover events [4]. With increasing human–wildlife interactions, particularly through live animal markets and agricultural practices, zoonotic influenza viruses have more opportunities to circulate, evolve, and potentially acquire characteristics that enhance human infectivity [5].

Avian influenza viruses (AIVs) have infected an extensive range of species beyond domestic poultry, including wild bird populations, marine mammals, and terrestrial mammals. The virus has had a devastating impact on wild birds, causing mass mortality events across migratory pathways and threatening biodiversity in affected regions [6]. Outbreaks have also been reported in fur farms, particularly among mink and foxes [7]. HPAI has been detected in dairy cattle herds, raising concerns about new transmission routes and increased opportunities for viral adaptation [8]. Human cases, though relatively rare, have been documented and often linked to close contact with infected animals or contaminated environments [9].

The dynamics of zoonotic influenza transmission are complex, involving multiple species and various direct and indirect pathways. Wild birds, especially migratory species, play a critical role in the dissemination of AIVs across geographical regions [10]. Migratory routes can facilitate the spread of influenza viruses over long distances, leading to outbreaks in previously unaffected regions [11]. Waterbirds are known to be the natural reservoirs of influenza A viruses [12]. When these wild species interact with domestic animals, such as poultry, at shared water sources or through direct contact, cross-species transmission of influenza viruses becomes more likely [13]. The role of domestic animals in influenza transmission is also crucial, particularly within agricultural systems where biosecurity measures are limited. In such settings, poultry may act as intermediate hosts, facilitating the transmission of influenza viruses from wild avian species to humans [14]. Vehicles, equipment, and personnel moving between farms can further spread influenza viruses, underscoring the importance of biosecurity and sanitation protocols [15]. Human infection with zoonotic influenza viruses typically occurs through direct contact with infected animals or indirectly via contaminated environments in the animal growing sector with the risk increasing in lower biosecurity settings such as backyard growing, live bird markets (LBMs), and agricultural fairs. LBMs, in particular, are hotspots for zoonotic transmission, with high viral loads maintained in the environment due to inadequate sanitation and biosecurity measures [16].

Environmental factors such as climate, water sources, and seasonal migrations of wild birds influence influenza virus survival and spread [17]. Wild bird habitats, such as wetlands, are particularly important, as these environments attract migratory birds and allow influenza viruses to persist in water and potentially spread to other species [12]. Climate factors like temperature and humidity can also impact influenza virus stability [18]. Genetic factors within influenza viruses also influence their potential for zoonotic transmission. Influenza A viruses, particularly those of avian origin, have demonstrated the ability to acquire mutations that enhance their infectivity and transmissibility in mammalian hosts [3].

Given the multifaceted nature of zoonotic influenza transmission, understanding the combined effects of environmental conditions, host interactions, and viral adaptations is essential for effective surveillance and control. WHO’s Global Influenza Programme developed the WHO public health research agenda for influenza in 2009, highlighting the importance of research in influenza prevention and control to enhance global knowledge, preparedness, and response capabilities. The agenda was reviewed in 2010–2011 and then in 2017 to guide researchers and outline directions and priority areas for research for the next 5–10 years. This agenda recommended research to further define the host-to-host transmission pathways of influenza A viruses from animal to animal, and from animals or animal environment to humans, especially by occupational exposure. Additionally, other human-related factors were shown to contribute to influenza virus infection [19,20,21]. Since 2017, research has been conducted, and major changes have occurred to the global health landscape, impacted mainly by the COVID-19 pandemic. Therefore, there is a need to review the transmission pathway studies conducted during the last eight years across different geographic regions and animal species and analyze their findings.

We conducted a comprehensive literature review to highlight recent research findings from 2017 to 2024, focusing on the primary zoonotic influenza virus transmission pathways, factors influencing zoonotic transmission, and key knowledge gaps. We evaluated data from studies on wild and domestic animal transmission, host and environmental factors, and transmission pathways studies in experimental settings.

## 2. Materials and Methods

### 2.1. Search Strategy

**Figure 1:**
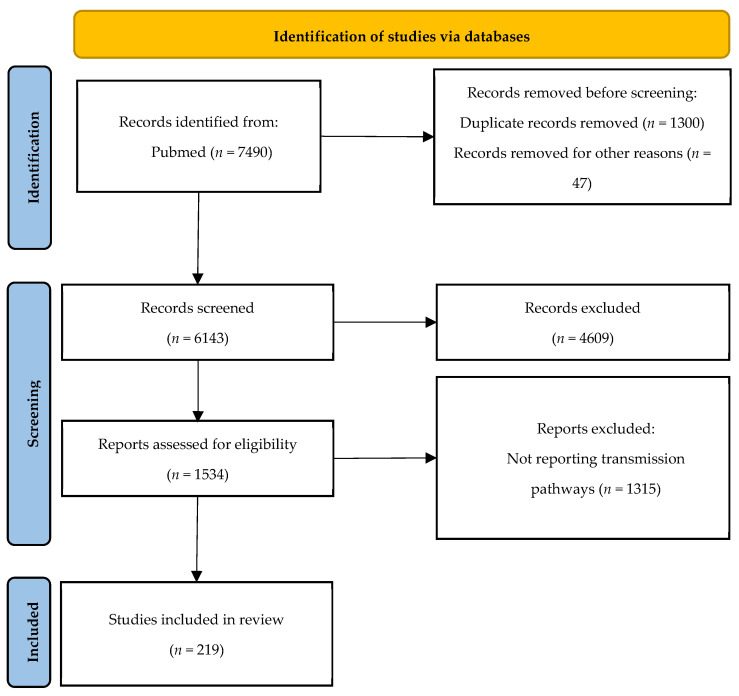
PRISMA flow diagram of the screening and selection of studies for this review.

This study was designed following the Preferred Reporting Items for Systematic Reviews and Meta-Analyses (PRISMA) 2020 for review of the published peer reviewed literature. This study did not require institutional review board approval. We used PubMed to search for peer-reviewed publications using the search terms “avian influenza”, “swine influenza”, and “zoonotic influenza viruses” from 2017 to 2024 (Figure 1). In total, 7490 records were exported to an Endnote X8 (Endnote, Berkley, CA, USA) library. Duplicates and papers published after 31 March 2024 were removed, yielding 6143 publications retained and exported to a master Excel (Version 2408) spreadsheet (Microsoft, Redmond, WA, USA). The following data were extracted from each included study: publication year, country, and category. To select the papers that discussed transmission pathways of zoonotic influenza, we searched for the keywords “transmi” in the master Excel spreadsheet. The search yielded 1534 records. Titles and abstracts were reviewed thoroughly and were independently screened by two researchers to select only the papers following the eligibility criteria listed in Section 2.2. A total of 219 research papers were included in this review. The selection was confirmed by a senior scientist with extensive expertise in public health and more than 10 years of experience in zoonotic influenza.

### 2.2. Eligibility Criteria

The included publications met the following eligibility criteria: (1) publications reporting direct and indirect transmission pathways between wild animals, between wild and domestic animals and among domestic animals, and between animals and humans; publications discussing the role of the environment and companion animals in the transmission of zoonotic influenza; publications discussing host, virus, and environmental factors influencing transmission of zoonotic influenza; and publications discussing experimental transmission settings; (2) publications are published in the period from 2017 to end of March 2024. Publications were excluded for the following reasons: (1) duplicates, (2) published after 31 March 2024, (3) records not reporting transmission pathways and related topics.

### 2.3. Data Collection Process

Two researchers collected data from the 219 retained peer-reviewed publications independently to generate a literature review. Extracted data were reviewed by the senior scientist. We extracted information on direct and indirect transmission pathways involving wild animals, transmission between wild and domestic animals and among domestic animals, and transmission between animals and humans. Additionally, we reviewed the role of environmental factors and companion animals in the spread of zoonotic influenza, along with factors influencing transmission dynamics. Experimental transmission settings to better understand these interactions were also examined.

## 3. Findings

### 3.1. Direct and Indirect Zoonotic Influenza Transmission Pathways Among Wild Animals

Studies showed how wild birds significantly contribute to the spread of influenza viruses. In South Korea, a case report of HPAI H5N8 virus infection in a common pochard attacked by a bird of prey suggests potential transmission of HPAI from infected waterfowl to predators [22], with cross-species transmission among wild bird species contributing to the ongoing circulation of HPAI H5N1 clade 2.3.4.4b in wild bird populations and its further geographic spread through migratory movements [23]. Migratory Whooper Swans were shown to be key vectors for spreading the H5N1 virus between China and Mongolia [24], and migratory stopover sites in the U.S. were shown to play a key role in AIVs spread among migratory birds, highlighting the role of migration in the epidemiology of the spread of AIVs [25]. In Spain, the composition and size of breeding Anseriformes populations were strongly associated with the circulation and maintenance of avian influenza viruses [26]. Infections of mammalian species with HPAI H5N1 have been reported from the Netherlands in three wild red foxes, probably due to foxes feeding on infected birds, and phylogenetic analysis showed that the viruses were related to HPAI H5N1 clade 2.3.4.4b found in wild birds [27]. Furthermore, a spillover of H5N1 into marine mammals was reported from the northeastern U.S., resulting in unusual mortality in seals [28]. Similarly, a massive outbreak of H5N1 clade 2.3.4.4b occurred in 2023 in elephant seals in Argentina, with ecological and phylogenetic data supporting mammal-to-mammal transmission [29].

### 3.2. Direct and Indirect Zoonotic Influenza Transmission Pathways Between Wild and Domestic Animals

Findings highlighted that migratory patterns, especially in regions where wild and domestic bird habitats overlap, like the Black-sea Mediterranean region, increase the risk of influenza virus spread and reassortment [30]. Wild birds were shown to play a central role in introducing and transmitting AIVs to poultry across various regions, including China [31], Europe, [32], South Korea, and Japan [33]. Wild waterfowl migration routes, often passing through wetland and livestock facilities, provide direct pathways for AIVs transmission into domestic poultry [34]. Wild birds, both sedentary and migratory, have been identified as key reservoirs and intermediaries for avian influenza transmission to poultry [35,36], with risk increased by contaminated inland waters and shared food resources close to poultry farms through direct contact or by attracting wild birds that serve as intermediary reservoirs [37]. For instance, wild waterfowl were shown to have introduced H5N8 HPAI into domestic ducks [38]. In the Czech Republic, H5N8 infections in poultry were linked to wild birds, spreading through both direct contact and indirectly via movement of infected materials and contaminated equipment [39]. Wild waterfowl have also been implicated in HPAI H5N6 outbreaks in Japanese zoo birds [40]. Certain practices, such as supplemental feeding that attracts more wild birds increasing wild bird-chicken interactions [41], free-grazing practices that expose ducks to wild birds and contaminated environments, transportation of ducks by carrying infected ducks and contaminated materials from one site to another [42], and the trade of live-trapped wild birds at live poultry markets [43], increase the risk of AIVs transmission through both direct and indirect pathways. In October 2022, an H5N1 outbreak occurred at a mink farm in northwest Spain housing more than 50,000 minks, with wild birds likely playing a major role in the virus’s introduction [44]. HPAI H5N1 was detected on 27 fur farms in Finland in July 2023, including silver and blue foxes, American minks, and raccoon dogs [7].

### 3.3. Direct and Indirect Zoonotic Influenza Transmission Pathways Among Domestic Animals

Studies have shown that various practices in poultry and duck farming significantly impact the spread of avian influenza viruses. Vehicle movements between farms, live poultry trading networks, and poor transport practices contribute to the spread and genetic diversification of avian influenza viruses such as H5N6 and H7N9, highlighting the need for stringent biosecurity and sanitation measures [15,45,46]. Semi-scavenging ducks and connected duck farms through physical interaction or through shared resources can act as reservoirs and key nodes in the spread and genetic diversification of avian influenza viruses by facilitating transmission between wild and domestic avian populations [47,48]. The recent outbreaks of HPAI H5N1 in cattle and goats in the U.S. raise significant global health concerns, underscoring the virus’s ability to cross unconventional host interfaces and further expand its host range [49]. Interestingly, infected cows were shown to shed virus in raw milk and cow-to-cow transmission potentially occurred, as infections were detected on farms where cows infected with AIVs had been transported [50]. Moreover, in April 2024, deceased cats on a dairy farm with H5N1-positive cows in the U.S. were shown to be infected with H5N1, likely from consuming unpasteurized milk from infected cows. These findings suggest increasing virus adaptation in mammals [50].

### 3.4. Direct and Indirect Zoonotic Influenza Transmission Pathways Between Animals and Humans

Studies have identified several zoonotic risks for influenza transmission into humans. In Peru, poultry and pork production settings harbor zoonotic pathogens with poor hygiene, improper handling, cross-contamination between different types of meat and between animals and humans, and inadequate use of personal protective equipment contributing to transmission risks from animals to humans [51]. The risk of LPAI transmission from raw eggs or poultry meat is negligible for commercial poultry and humans exposed through consumption and very low for non-commercial poultry, wild birds, and those handling raw products [52]. Additionally, semiaquatic mammals could act as intermediate hosts for AIVs, suggesting that these animals could be important targets for surveillance and control efforts [53].

#### 3.4.1. Transmission of Avian Influenza H7Nx Between Animals and Humans

Studies on H7 AIV revealed several risk factors for human infection. One study linked a human H7N9 infection to improper poultry processing, with genetic analysis suggesting that the virus could be highly pathogenic with the insertion of multiple basic amino acid residues (PEVPKRKRTAR/GL) at the HA cleavage site [54]. In another case, two family members were infected with H7N9 in China after exposure to sick chickens in a poultry market, with the virus showing avian and human dual-receptor specificity [55]. Exposure to live poultry markets, particularly sick or dead poultry, increased infection risks [56,57]. Market closure in China led to a significant decrease in human H7N9 infections [58]. City-wide closures and extending or permanently closing markets could further help manage avian influenza risks [59,60]. Following outbreaks of LPAI H7N2 in cats in animal shelters in New York, the virus was detected in both air and surface samples collected from a feline quarantine facility, indicating risk for contact and airborne transmission of the virus to emergency response workers [61].

#### 3.4.2. Transmission of Avian Influenza H5Nx Between Animals and Humans

Recent studies underscore the risks of H5 AIV transmission between animals and humans. In England, a case of asymptomatic H5N1 infection in a duck owner highlights the importance of active surveillance of asymptomatic exposed individuals [62]. Research in Chile identified mutations in the PB2 gene of HPAI H5N1 viruses that enhance infection in mammalians [63]. Additionally, a fatal H5N1 infection was reported in a pregnant woman after contact with sick poultry [64], while another human case with avian-origin H5N6 arose from exposure to slaughtered poultry, highlighting an important route of transmission from birds to humans [65]. Reports also indicate avian-to-swine transmission of H5N1 and H5N8 viruses and swine-to-human transmission of IAV, highlighting the importance of surveillance of IAV in swine [66]. A study in China linked H5N6 spread to poultry movement and trade, emphasizing the role of live poultry movement in AIV transmission [67]. Human infections with HPAI were reported. On 29 March 2023, the first confirmed human infection with H5N1 was reported in Chile to WHO, likely transmitted through environmental exposure near the person’s residence, where sick or dead sea mammals or wild birds were found [68]. In late March 2024, a dairy farm worker in Texas was infected with HPAI H5N1 after direct, close contact with sick cows, showing symptoms consistent with confirmed H5N1 cases in other nearby farms in northern Texas [69].

#### 3.4.3. Transmission of Avian Influenza H9N2 Between Animals and Humans

Although generally, H9N2 viruses are considered as the LPAI viruses in birds [70], more than 100 laboratory-confirmed human H9N2 infections have been reported from WHO [71]. Notably, over 50 cases occurred after the COVID-19 outbreak. Human infections were linked to environmental contamination in LBMs [72], where air samples from these markets, such as in Chinese LBMs, revealed H9N2 strains with molecular traits related to enhanced pathogenicity to humans, indicating potential airborne spread of the virus within the market environment and potential transmission to humans [73]. In addition, direct contact with infected poultry, especially among occupational groups, significantly increases infection risks through respiratory droplets or contaminated surfaces [74,75,76]. A seroprevalence study in China between 2014 and 2016 revealed that 11.2% of healthy occupational workers have antibodies against H9N2 AIVs [77]. The propensity of H9N2 to reassort with other avian influenza viruses has been documented, enhancing viral adaptability and cross-species transmissibility [78,79,80]. Furthermore, unlike HPAI H5N1 human infection cases, the mild nature of H9N2 human infection has often led to their clinical oversight, allowing the virus to adapt within the host through reassortment with other human seasonal influenza viruses, potentially giving rise to highly replicative variants with efficient human-to-human transmissibility.

#### 3.4.4. Transmission of Swine Influenza Virus Between Animals and Humans

Three subtypes of swine influenza A viruses (IAV-S) circulate globally (H1N1, H1N2, and H3N2). Studies have shown multiple factors contributing to the transmission of IAV-S between animals and humans. In Germany, an H1N1 IAV-S infection in a child was traced to contact with pigs, and the isolated virus was antigenic close to A(H1N1)pdm09 [81]. In the U.S., swine shows have been identified as hotspots for IAV-S spread among pigs and spillovers into humans, with shorter exhibition periods and postponing show schedules suggested to reduce IAV-S transmission [82,83]. Agricultural fairs pose an additional risk, with IAV-S recovered from both the air and surfaces, indicating environmental contamination [84]. Close contact between pigs and farm workers [85], along with certain swine management practices during movement of exhibition swine, such as biosecurity measures, housing conditions, and movement protocols, also increase IAV-S transmission risk, emphasizing the need for preventive measures at interconnected swine events [86,87].

#### 3.4.5. Reverse Influenza Virus Transmission from Humans to Animals

Human are considered as potential “mixing vessels” hosts for the generation of zoonotic animal influenza viruses, and studies have documented various instances of reverse zoonotic transmission of influenza viruses from humans to animals. However, so far, no study has reported the transmission of zoonotic influenza viruses to animals. Several studies have shown the transmission of human seasonal influenza viruses to animals, in particular to swine. In one case, the seasonal human A(H1N1)pdm09 virus was transmitted from caregivers to cheetahs in a zoo, resulting in subsequent cheetah-to-cheetah transmission [88]. Human seasonal H3N2 influenza was transmitted to and became endemic in swine populations, posing a risk of re-transmission to humans [89]. Additionally, a first case of influenza B transmission from domestic pigs to humans by droplets or close contact was reported, suggesting that domestic pigs have been previously infected with influenza B virus from humans [90]. Human-to-swine transmission of pandemic A(H1N1)pdm09 virus was reported globally [66]. Poor husbandry and biosafety practices of farmers at the human–swine interface facilitate reverse zoonotic transmission [91]. Studies in Japan and West Africa have highlighted bidirectional transmission of A(H1N1)pdm09 virus between humans and swine, emphasizing the importance of surveillance and preventive measures to manage zoonotic and reverse-zoonotic transmissions [92,93]. Pigs were shown to play a significant role in interspecies transmission of both seasonal and zoonotic influenza viruses [94].

### 3.5. Host, Virus, and Environmental Factors Influencing Transmission

#### 3.5.1. Environmental Factors Influencing Transmission

Farm-level risk factors influencing influenza transmission were reported. In Japan, large flock sizes and proximity to water bodies increase the risk of HPAI infection on chicken farms, as does frequent access of farm personnel and equipment to premises and waterfowl visiting nearby water bodies in large farms [95]. In Bangladesh, inadequate biosecurity measures, poor sanitation, and close contact between chickens and other poultry or wild birds showed to increase the likelihood of H5 and H9 seropositivity [96]. Weather patterns, including temperature, humidity, and precipitation, significantly influenced the spread and transmission of HPAI and AIVs, with factors like average daily maximum temperature, the global solar radiation, wind spread, ocean temperatures, and characteristics of water bodies playing key roles in various regions including Japan, Bangladesh, China, the U.S., and Germany [17,97,98,99,100]. Market and trade-related practices in LBMs, such as poor hygiene, overcrowding, mixing different poultry species, contaminated transport vehicles, and inadequate biosecurity measures across the supply chain increase the risk of AIVs transmission, particularly in Vietnam, Bangladesh, and Iran [101,102,103], where these markets serve as key sites contributing to the mixing and reassortment of influenza viruses and the emergence of more virulent strains that can cross species barriers and infect humans [42,43,104,105,106]. Migratory birds play a major role in long-distance AIVs transmission. In the U.S., bird migration can transport AIVs over long distances, and breeding habitats of migrant species increase viral reassortment [17], while in Asia and Africa, H9N2 virus was shown to spread over long distances through migratory birds and over short distances via poultry trade and human activity [107]. Additionally, areas with high AIVs contamination correlated with higher human infection rate, underscoring the need for improved environmental conditions to reduce the risk of human infections [108]. High-density production systems, such as mink and dairy farming, pose significant risks for future viral pandemics. The intensive mink farming conditions, where mink are kept in high-density environments, facilitate rapid viral spread and evolution, increasing the likelihood of zoonotic spillover events [109]. Notably, mink have been involved in outbreaks of SARS-CoV-2 [110] and HPAI H5N1 [44], demonstrating their capacity to act as intermediary hosts for viruses that can infect humans. Keeping large numbers of animals in crowded, stressful conditions increases the risk of pathogen emergence and spread, including zoonotic diseases with pandemic potential [111].

#### 3.5.2. Virus Factors Influencing Transmission

Many studies have discussed the emergence of viruses with mammalian adaptation markers, increased polymerase activity in mammalian cells, and enhanced receptor binding preference for human-like receptors [112]. H9N2 viruses with tribasic amino acid residues at the hemagglutinin cleavage site were shown to have increased viral replication, stability, pathogenicity, and transmission in chickens compared to H9N2 with monobasic amino acid residue at the hemagglutinin cleavage site [113]. Several mutations were identified to be associated with human adaptation of AIVs. In H5 AIVs, 102 adaptive evolution sites in 8 genes were reported [114]. Minor alterations in the amino acid sequence of the HA and/or the combination of H9N2 surface genes with internal genes of human influenza viruses allowed new H9N2 viruses to transmit via aerosol [115]. The concurrent presence of two AIVs, H5N1 and H9N2, in Egypt was shown to have important implications on public health due to increased risk of human exposure. The interaction between the two viruses may enhance the potential of genetic reassortments and emergence of more virulent strains. For instance, H5N1 viruses acquired mutations that enhance transmissibility and replication in mammalian hosts. H9N2 viruses acquired mutations that increase human-like receptor specificity [116]. Avian H4N6 isolate containing Q226 and G228 mutations preferentially bound to avian receptors, whereas swine H4N6 isolate containing L226 and S228 preferentially bound to human receptors [117]. Four accumulated mutations in PA of a swine influenza reassortant virus A/swine/Liaoning/265/2017 (H1N1) (LN265) caused increased pathogenicity of the virus in mice and transmissibility in ferrets [118].

#### 3.5.3. Host Factors Influencing Transmission

The polymerase of avian influenza A viruses catalyzes the replication and transcription of the virus but has poor performance in mammalian cells due to the acidic nuclear phosphoproteins of 32 kDa (ANP32) protein, a host factor responsible for regulating virus replication and restricting AIV polymerase activity. However, swine ANP32A with two key sites, 106V, unique to pigs, and 156S, was shown to enhance avian viral polymerase activity and allow the virus to replicate [119]. Mutations in residues 313 of the viral nucleoprotein (NP) of AIVs, such as H7 and H9, escaped human butyrophilin subfamily 3 member A3 (BTN3A3), an efficient inhibitor of AIVs expressed in human airways, which increases zoonotic potential of AIVs [120]. Feather epithelium in ducks was shown to be a significant contributor to the dissemination of H5N1 through the viral particles that can be detected in the feathers of infected ducks. In addition, feather epithelium showed to contain high viral loads. Hence, feather epithelium can spread the virus through direct and indirect contact with other birds and enhance environmental contamination, contributing to the maintenance of viral infectivity and to viral transmission over long distances between poultry farms [121].

### 3.6. Transmission of Zoonotic Influenza in Experimental Settings

More than 50 papers reported controlled laboratory studies to identify the mechanisms of AIVs transmission in birds, study pathogenicity, infectivity, and transmissibility, and monitor infection rates, virus shedding, mode of transmission, binding to different receptors, and the potential of different bird species to act as potential hosts for AIVs by stimulating different exposure scenarios [122,123,124,125,126,127,128,129,130,131,132,133,134,135,136,137,138,139,140,141,142,143,144,145,146,147,148,149,150,151,152,153,154,155,156,157,158,159,160,161,162,163,164,165,166,167,168,169,170,171,172,173,174,175,176,177,178,179,180,181,182,183,184,185,186,187,188,189,190]. These studies showed that several clades of H5Nx and H9N2 viruses have demonstrated varying levels of pathogenicity and transmissibility depending on host species and key mutations in viral proteins associated with increased virulence, enhanced replication, and adaptation to new species. The infectivity, antigenicity, replication, pathogenicity, and transmission of different avian influenza strains have been studied in mice to identify their pathogenicity in mammals [191,192,193,194,195,196,197] and showed key mutations linked to increased replication and host adaptation in mammals. Few papers reported the experimental transmission of avian influenza in swine [198,199,200,201,202,203,204,205] and showed that certain viruses can infect swine with varying degrees of replication and transmission potential. Since dogs may act as an intermediate host for AIVs and increase the likelihood of zoonotic transmission of influenza viruses, experimental transmission of AIVs to dogs and from dogs was conducted and reported in two studies [206,207]. Few papers investigated the transmissibility and pathogenicity of AIVs in chickens, BALB/c mice, and guinea pigs [208,209] and showed that H3N8 viruses acquired enhanced pathogenicity and replication in mammals following adaptation in mice while the novel H7N6 reassortant virus was capable of efficient respiratory droplet transmission in guinea pigs. A study was conducted to understand the barrier against influenza emergence in horses despite frequent transmission from wild birds to horses and showed the lack of adaptive evolution of the viruses in the equine host [210]. Experimental transmission of avian influenza viruses in ferrets was also reported [197,211,212,213,214,215,216,217,218,219,220,221,222,223,224,225,226,227,228,229,230,231], as well as an H7N9 virus isolated from a human that was lethal in ferrets via respiratory droplets, an H5N6 virus leading to severe multi-organ pathology in ferrets, and some mutations in HA and PB1 that enhanced receptor binding, virulence, and mammal-to-mammal spread. Experimental transmissions of influenza B and D in pigs and calves were conducted to elucidate species-specific replication and transmission dynamics [232,233,234] and showed that influenza D replicated more efficiently and transmitted better than influenza B in pigs, suggesting stronger adaptation and that influenza D is capable of aerosol transmission between cattle and pigs. The pathogenicity and transmissibility of canine influenza was studied in mouse and guinea pig models [235,236], in ferrets [237,238], and in domestic poultry [239] to investigate their susceptibility to canine influenza A virus and showed that certain isolates replicated efficiently in mammals, with some mutations linked to enhanced adaptation and airborne spread. A study showed that a single amino acid change significantly impacts the transmissibility of H1N1 IAV-Ss in guinea pigs, serving as a model for human infection [240]. Experimental transmission studies of swine influenza virus in ferrets were reported [241,242,243,244,245,246,247,248,249,250,251,252,253]. These studies showed that HA stabilization and enhanced polymerase activity increased pathogenicity and droplet transmission. Some reassortant swine influenza viruses were capable of transmitting from pigs to ferrets and showed poor cross-reactivity with current human vaccine strains, underscoring their zoonotic potential.

## 4. Discussion

This review has identified several gaps in the current research on zoonotic influenza transmission, underscoring the need for a more comprehensive approach to understand, prevent, and manage the risks posed by those viruses. Although numerous studies have focused on specific regions or species, research remains limited in several critical areas including transmission pathways among diverse animal populations, the role of environmental factors, and the zoonotic potential of influenza viruses across different geographic contexts. Addressing these gaps is essential for improving public health strategies that aim at mitigating the risks of zoonotic influenza outbreaks.

Studies on direct and indirect transmission pathways remain regionally limited and concentrated on specific species. Most research has focused on migratory wild birds as primary reservoirs of AIVs. However, there is a shortage of comprehensive studies investigating the full range of direct and indirect transmission routes involving both wild and domestic animals. For instance, a few studies explore the interaction zones where wild birds, domestic poultry, and other potential hosts, like semi-aquatic mammals, come into contact. These zones could be critical for understanding cross-species transmission dynamics, as they often present high-risk conditions for spillover events. Expanding research on these pathways across diverse ecosystems, especially in high-risk interface zones, would help clarify how influenza viruses are maintained and transmitted within and between animal populations. Although several studies have examined AIVs transmission between wild and domestic animals, research is limited in scope and tends to focus on poultry farms with limited biosecurity measures. Few studies provide detailed analyses of how specific practices in domestic animal production may increase interaction rates between wild birds and domestic poultry, thereby enhancing the risk of viral spillover.

Research to investigate the role of companion animals in the transmission of influenza viruses is sparse, with few studies examining how pets and other domestic animals may contribute to the spread of zoonotic influenza within household or urban settings. Expanding research in this area could provide insights into additional transmission pathways, particularly in cases where reverse zoonotic transmission from humans to animals has already been documented.

Additionally, research examining how environmental conditions and production practices in rural or urban settings influence transmission among domestic animals is sparse. In-depth studies on production practices that facilitate zoonotic influenza transmission between wild and domestic animals should be conducted. Targeted research on biosecurity practices in domestic farms, particularly in regions with high bird migration, could inform more effective interventions and guidelines for farm operations to reduce interspecies contact. Research on environmental factors, including climate, seasonality, and proximity to water sources, is limited, particularly studies examining the role of climate patterns on virus persistence and spread in both wild and domestic populations. Further, limited research has been conducted on how urbanization and agricultural expansion might impact zoonotic transmission in different geographic areas. Conducting studies on the impact of climate and environmental factors on influenza virus transmission, especially in varied ecosystems, would enhance understanding of outbreak risks.

Studies on viral mutations and host factors that increase the zoonotic potential of influenza viruses are still sparse. Specific mutations that enhance receptor binding affinity to mammalian cells have been observed, but there is a limited understanding of how these adaptations influence zoonotic transmission across different species. Additionally, host-specific factors, such as genetic susceptibility and immune responses, are under-researched, particularly in swine and semi-aquatic mammals that may act as intermediaries between avian species and humans. Studies on the role of intermediate hosts in the zoonotic transmission of influenza viruses, species-specific adaptations, host immune system and interference with pre-existing immunity either from prior infection or from vaccination, host genetic susceptibility, and the potential role of viral genetic mutations in cross-species transmission should be conducted.

Experimental transmission studies have contributed valuable insights into the pathogenicity and transmissibility of various influenza strains. However, the lack of standardization in laboratory experiments limits comparability across studies. Standardizing laboratory experiment designs in zoonotic influenza research would improve the comparability and reproducibility of findings. Such standardization would allow for more cohesive insights into zoonotic influenza dynamics across different research settings.

Research has shown that LBMs often host multiple influenza strains, posing risks for interspecies transmission and virus reassortment. However, due to regional restrictions, continuous monitoring is limited, which hinders the timely detection of emerging strains. Additionally, asymptomatic individuals who are exposed to avian influenza in high-risk settings, such as LBMs or poultry farms, are underrepresented in current surveillance efforts, limiting understanding of human infection risks. Including asymptomatic exposed individuals in surveillance programs would improve public health risk assessments and case management, enabling earlier detection of zoonotic transmission potential.

## 5. Conclusions

This systematic review highlights the multifaceted dynamics of zoonotic influenza transmission. The findings emphasize that migratory birds and live animal markets play central roles in virus dissemination, serving as significant nodes for cross-species transmission. Additionally, environmental conditions, such as proximity to water bodies and climate variability, significantly influence viral survival and transmission risks across diverse geographic regions. While much progress has been made in understanding these pathways, critical research gaps remain, particularly concerning transmission routes involving companion animals and intermediate hosts, environmental drivers, and the role of genetic adaptations in increasing zoonotic potential.

To enhance our preparedness and response to zoonotic influenza threats, targeted surveillance and research efforts are needed in high-risk areas, such as live bird markets and wildlife–livestock interface zones. Implementing consistent biosecurity protocols, alongside early detection systems and genetic monitoring, can help mitigate the risk of zoonotic spillovers and facilitate timely interventions. A One Health approach, which integrates human, animal, and environmental health, is essential for addressing the complexity of zoonotic influenza and for developing effective strategies that protect both public health and biodiversity.

## Data Availability

All data are present in the manuscript.
